# A Controlled Human Infection Model of Group A *Streptococcus* Pharyngitis: Which Strain and Why?

**DOI:** 10.1128/mSphere.00647-18

**Published:** 2019-02-13

**Authors:** Joshua Osowicki, Kristy I. Azzopardi, Liam McIntyre, Tania Rivera-Hernandez, Cheryl-lynn Y. Ong, Ciara Baker, Christine M. Gillen, Mark J. Walker, Pierre R. Smeesters, Mark R. Davies, Andrew C. Steer

**Affiliations:** a Tropical Diseases, Murdoch Children’s Research Institute, Melbourne, Victoria, Australia; b Department of Paediatrics, University of Melbourne, Melbourne, Victoria, Australia; c Infectious Diseases Unit, Department of General Medicine, The Royal Children’s Hospital Melbourne, Melbourne, Victoria, Australia; d Department of Microbiology and Immunology, The University of Melbourne, at the Peter Doherty Institute for Infection and Immunity, Melbourne, Victoria, Australia; e School of Chemistry and Molecular Biosciences and Australian Infectious Diseases Research Centre, The University of Queensland, St. Lucia, Queensland, Australia; f Paediatric Department, Academic Children Hospital Queen Fabiola, Université Libre de Bruxelles, Brussels, Belgium; g Molecular Bacteriology Laboratory, Université Libre de Bruxelles, Brussels, Belgium; UMKC School of Medicine

**Keywords:** *Streptococcus pyogenes*, controlled human infection model, group A *Streptococcus*, human challenge study, pharyngitis, vaccines

## Abstract

Group A *Streptococcus* (GAS) is a major cause of global infection-related morbidity and mortality. A modern controlled human infection model (CHIM) of GAS pharyngitis can accelerate vaccine development and pathogenesis research. A robust rationale for strain selection is central to meeting ethical, scientific, and regulatory requirements. Multifaceted characterization studies were done to compare a preferred candidate *emm*75 (M75) GAS strain to three other strains: an alternative candidate *emm*12 (M12) strain, an M1 strain used in 1970s pharyngitis CHIM studies (SS-496), and a representative (5448) of the globally disseminated M1T1 clone. A range of approaches were used to explore strain growth, adherence, invasion, delivery characteristics, short- and long-term viability, phylogeny, virulence factors, vaccine antigens, resistance to killing by human neutrophils, and lethality in a murine invasive model. The strains grew reliably in a medium without animal-derived components, were consistently transferred using a swab method simulating the CHIM protocol, remained viable at −80°C, and carried genes for most candidate vaccine antigens. Considering GAS molecular epidemiology, virulence factors, *in vitro* assays, and results from the murine model, the contemporary strains show a spectrum of virulence, with M75 appearing the least virulent and 5448 the most. The virulence profile of SS-496, used safely in 1970s CHIM studies, was similar to that of 5448 in the animal model and virulence gene carriage. The results of this multifaceted characterization confirm the M75 strain as an appropriate choice for initial deployment in the CHIM, with the aim of safely and successfully causing pharyngitis in healthy adult volunteers.

**IMPORTANCE** GAS (Streptococcus pyogenes) is a leading global cause of infection-related morbidity and mortality. A modern CHIM of GAS pharyngitis could help to accelerate vaccine development and drive pathogenesis research. Challenge strain selection is critical to the safety and success of any CHIM and especially so for an organism such as GAS, with its wide strain diversity and potential to cause severe life-threatening acute infections (e.g., toxic shock syndrome and necrotizing fasciitis) and postinfectious complications (e.g., acute rheumatic fever, rheumatic heart disease, and acute poststreptococcal glomerulonephritis). In this paper, we outline the rationale for selecting an *emm*75 strain for initial use in a GAS pharyngitis CHIM in healthy adult volunteers, drawing on the findings of a broad characterization effort spanning molecular epidemiology, *in vitro* assays, whole-genome sequencing, and animal model studies.

## Abstract (precis)

GAS (Streptococcus pyogenes) is a leading global cause of infection-related morbidity and mortality. A modern CHIM of GAS pharyngitis could help to accelerate vaccine development and drive pathogenesis research. Challenge strain selection is critical to the safety and success of any CHIM and especially so for an organism such as GAS, with its wide strain diversity and potential to cause severe life-threatening acute infections (e.g., toxic shock syndrome and necrotizing fasciitis) and postinfectious complications (e.g., acute rheumatic fever, rheumatic heart disease, and acute poststreptococcal glomerulonephritis). In this paper, we outline the rationale for selecting an *emm*75 strain for initial use in a GAS pharyngitis CHIM in healthy adult volunteers, drawing on the findings of a broad characterization effort spanning molecular epidemiology, *in vitro* assays, whole-genome sequencing, and animal model studies.

## INTRODUCTION

Group A *Streptococcus* (GAS; Streptococcus pyogenes) is a major contributor to global infection-related mortality and morbidity. It causes a diverse spectrum of human disease syndromes, from superficial infections (e.g., pharyngitis and impetigo) to invasive disease (e.g., necrotizing fasciitis and toxic shock syndrome) and autoimmune complications (acute rheumatic fever, rheumatic heart disease, and glomerulonephritis) (1). Development of a GAS vaccine has been impeded by scientific, regulatory, and commercial obstacles (2). Controlled human infection models (CHIM) are increasingly assuming an important role for vaccine development (3, 4). Drawing on the record of historical CHIM studies that included 172 participants (5–7), a modern pharyngitis CHIM in healthy adult volunteers has been proposed as part of a reenergized global effort to accelerate GAS vaccine development (8). Selection of a thoroughly characterized strain is central for development of a GAS CHIM.

A successful CHIM requires that infection and/or symptomatic disease endpoints are reached reliably and safely and bear sufficient resemblance to a natural state to suggest generalizability. The diverse clinical and microbiological profile of GAS presents challenges for CHIM study design, especially strain selection. There are more than 200 different GAS *emm* types. This widely used classification system is based on one part of the gene encoding a single GAS antigen, the M protein. No other antigen has been as closely studied, and the concept of M protein type-specific immunity has been a cornerstone of GAS research. GAS is a highly adapted human pathogen, and the limitations of *in vitro* assays and animal models have been well described. After more than a century of research, fundamental aspects of pathogenesis and human immune protection against GAS remain unknown. These knowledge gaps are simultaneously an argument for building a CHIM and a source of uncertainty in conceiving its design.

A thorough and explicitly stated rationale for strain selection is an important step in minimizing potential harm to participants and maximizing scientific impact. We considered desirable characteristics in selecting an initial strain to establish a GAS pharyngitis CHIM and surveyed available collections for suitable strains, focusing on an *emm*75 strain (GAS M75 611024, termed M75) isolated in 2011 from the throat of a 5-year-old girl with acute symptomatic pharyngitis in Melbourne (Table 1; see also Table S1 in the supplemental material) (9). In this paper, we present a multifaceted characterization of the preferred CHIM candidate M75 strain and compared it to three others: GAS M12 611025 (M12), an alternative challenge candidate; M1T1 5448 (5448), representative of the M1T1 clone recently responsible for most pharyngitis and invasive disease globally (10); and CDC SS-496 (SS-496), an M1 strain administered to 88 subjects in 1970s pharyngitis CHIM studies (5, 7).

**TABLE 1:** Preferred strain characteristics for a controlled human infection model of GAS pharyngitis^*a*^

Desirable strain characteristic(s)	Rationale	M75 611024 details
Definite but uncommon contemporary cause of symptomatic pharyngitis	Pharyngitis is the critical early target for GAS vaccine development; historical CHIM studies offer a template for a reliable and safe protocol; GAS pharyngitis is most common in childhood and adolescence, suggesting previous exposure and immune memory could prevent experimentally induced disease in adult volunteers	From a child with symptomatic GAS pharyngitis in Melbourne, 2011; preexisting immunity in adults is unknown (no correlate of protection); ≤5% of strains in most recent pharyngitis studies are *emm*75
Should cause skin infection	Common GAS skin infections (e.g., impetigo) will also be important in initial vaccine field trials; ideally, the pharyngitis CHIM strain(s) should also be suitable for use in a potential future human model of cutaneous GAS infection	E pattern generalist (throat and skin infections); cluster E6 is linked phylogenetically to D pattern skin isolates
Uncommon cause of invasive GAS disease and immunological sequelae	GAS pharyngitis can lead to locally invasive infectious complications (e.g., retropharyngeal abscess), severe invasive infection (e.g., streptococcal toxic shock syndrome), acute rheumatic fever, and glomerulonephritis	≤5% of isolates in recent reports of invasive GAS are *emm*75; from 2000 to 2016, 403/17,002 (2.4%) typeable invasive isolates reported to the U.S. CDC’s Active Bacterial Core surveillance were *emm*75 (Chris A. Van Beneden, personal communication, 11 September 2018) (41, 42); *emm*75 strains rarely associated with ARF/RHD or APSGN (1)
Should have predictable and limited virulence and be suitable for use in animal models	Whole-genome sequencing, *in vitro* assays, and animal models may inform understanding of a GAS strain’s relative virulence, although none fully predict human disease patterns	CovR/S virulence regulator, wild type (nonmutant); does not bind plasminogen and fibrinogen; *emm*75 strains have been used in animal nasopharyngitis and invasive disease models
Should have limited antibiotic resistance	Ideally, the challenge strain should be eradicated from the pharynx by antibiotic treatment; resistance to penicillin has not been documented in GAS, but it does not reliably eradicate GAS from the pharynx; observed resistance to other drugs is variable	See the text
Challenge strain should possess a wide array of candidate vaccine antigens	For greatest impact, a GAS pharyngitis CHIM should be suitable for early use as a preliminary testing ground for vaccines	See the text

a See Table S1 for a detailed and referenced version of this table. ARF, acute rheumatic fever; APSGN, acute poststreptococcal glomerulonephritis; RHD, rheumatic heart disease.

10.1128/mSphere.00647-18.1 TABLE S1 Preferred strain characteristics for a controlled human infection model of GAS pharyngitis. Download Table S1, PDF file, 0.2 MB. Copyright © 2019 Osowicki et al. 2019 Osowicki et al. This content is distributed under the terms of the Creative Commons Attribution 4.0 International license.

## RESULTS

### Growth in an animal-free medium.

**FIG 1:**
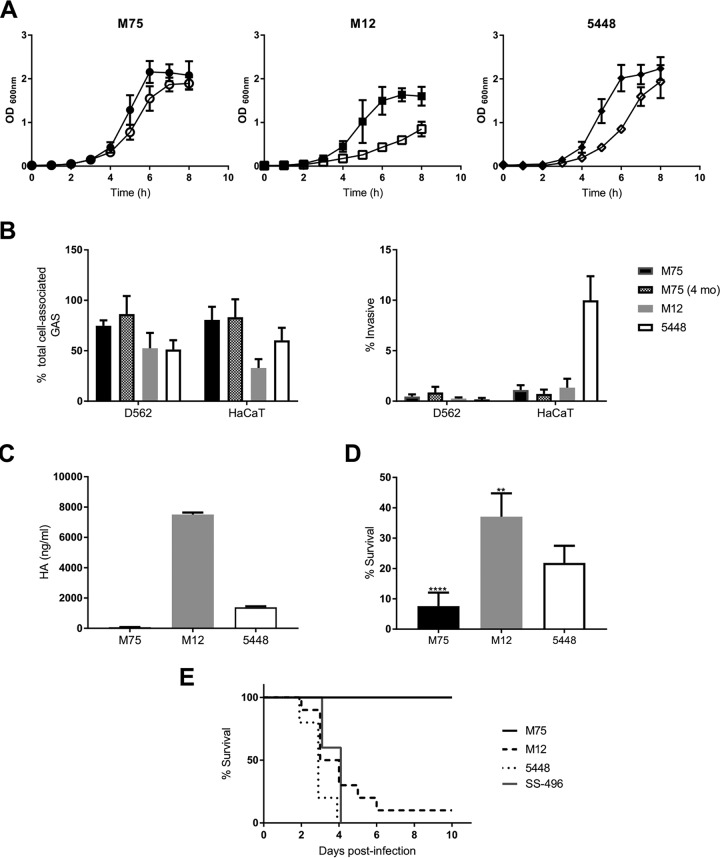
*In vitro* characterization of contemporary candidate strains for human challenge. (A) Growth kinetics of candidate strains in RPMI 1640 supplemented with 2% Veggietone (filled symbols) and Todd-Hewitt broth with 1% yeast extract (open symbols). Means and standard deviations (SD) are representative of three separate experiments done in triplicate. (B) Strain attachment and cellular invasion. Means and SD are from three separate experiments with triplicate wells. (C) Capsular hyaluronic acid quantification. Means and SD are derived from a single experiment. (D) Resistance of M75, M12, and 5448 to killing by human neutrophils. Means and SD are from three separate experiments using different blood donors, with seven biological replicates. (E) Strain lethality in a humanized plasminogen transgenic AlbPLG1 murine invasive disease model (*n* = 10 for each strain).

Compared to that in THY broth, no detrimental effect on growth of M75, M12, and 5448 was observed in the animal-free medium (Fig. 1A). Eight-hour growth curves for M75 clones tested after 7 days of repeated *in vitro* passage were similar to those of the nonpassaged parent (data not shown).

### Attachment properties.

M75 had the highest adherence to D562 (75%) and HaCaT (81%) cells (Fig. 1B). M12 (53%) and 5448 (51%) were similarly adherent to D562 cells. M12 preferentially adhered to D562 over HaCaT cells (P = 0.005), whereas M75 and M1T1 showed no preference. The affinity of M12 to pharyngeal over skin cells matches its designation as an A-C pattern strain, associated with throat tissue tropism (11). Invasiveness of M75 and M12 was low for both cell lines (≤0.45%). Invasion by 5448 of HaCaT cells (10%) was greater than that for D562 cells (0.2%) (Fig. 1B).

### Capsule production.

M75 produced 74 ng/ml of hyaluronic acid (HA) capsule, whereas M12 produced 7,506 ng/ml (Fig. 1C). Capsule production by 5448 matched previous findings (12).

### Delivery characteristics and viability.

**FIG 2:**
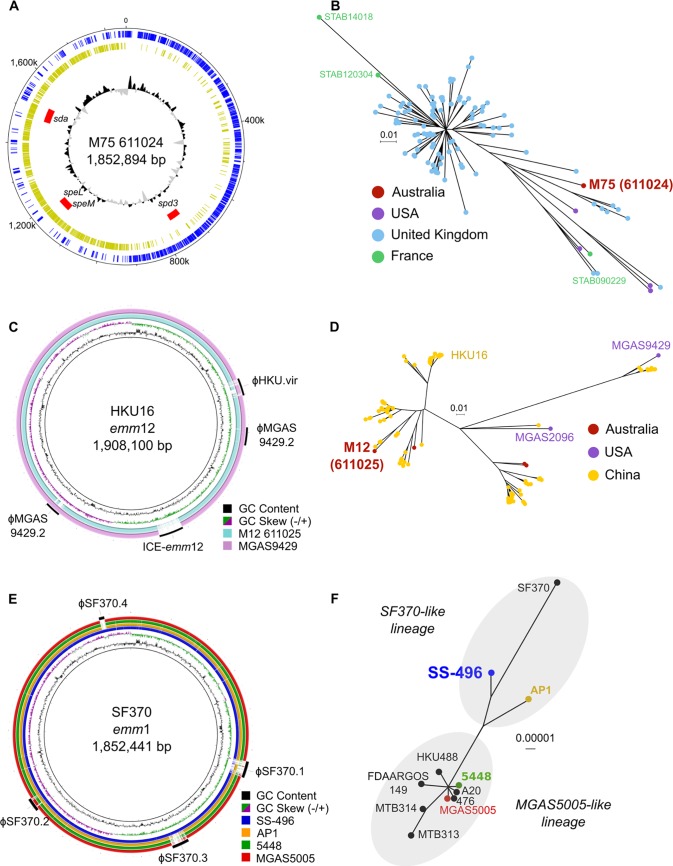
Comparative genomics of M75 611024, M12 611025, M1T1 5448, and M1 CDC SS-496. (A) Circular schematic of GAS M75 611024 showing GC plot (inner ring) with GC content above (black) and below (gray) the genome average. Predicted prophage sequences are shown in red, with associated prophage virulence determinants annotated and relative position of predicted coding sequences on the forward strand (blue) and reverse strand (gold). (B) Unrooted maximum likelihood tree of 131 *emm*75 strains from the United Kingdom, the United States, and France based on 1,046 SNPs relative to the M75 611024 reference genome. Tips of the tree are color coded based on country of isolation. Location of genomes corresponding to M75 611024 and the completely sequenced *emm*75 strains from France, STAB 090229 (CP020027), STAB 120304 (CP020082), and STAB 14018 (CP014542), are annotated. (C) Comparative BLASTN analysis of M12 611025 (blue ring) and MGAS9429 (purple ring) relative to the *emm*12 reference genome HKU16 (inner black circle). HKU16 GC content and GC skew are indicated in the inner ring, while annotated around the outside is the genomic position of known HKU16 mobile genetic elements. (D) Maximum likelihood phylogenetic relationship of strain 611025 with 141 *emm*12 S. pyogenes strains from other geographical regions based on 1,452 SNP sites from the core genome of the HKU16 reference genome. Tips of the tree are color coded based on country of isolation of each isolate. Genomes from completely sequenced *emm*12 strains MGAS9429 (CP000259), MGAS2096 (CP000261), and HKU16 (QMH11M0907901; AFRY01000001) are annotated. (E) Comparative BLASTN analysis of CDC SS-496 and other GAS M1 reference genomes, AP1, 5448, and MGAS5005, relative to the SF370 M1 GAS reference genome (inner black circle). (F) Mid-point-rooted maximum likelihood phylogenetic relationship of M1 GAS reference genomes based on 780 SNP sites. Tips of the tree are annotated by strain name and color coded by ring color from panel E. Genomes belonging to SF370-like and MGAS5005-like lineages (14) are clustered by gray shading. Comparative BLASTN analyses were generated using BRIG (40).

The Dacron swab was considered most suitable for delivery of the challenge inoculum (Fig. S1). Mean broth uptake by Dacron (105 mg) and Rayon S (108 mg) swabs was comparable to that of cotton (129 mg), and uptake variance was lowest (7.7 mg) for the Dacron swab. Superior release was noted for the Dacron swab with a mean of 1.8 × 10^3^ CFU of M75 recovered after swab dipping (Fig. 2B). Recovery from M75 vials frozen for 4 months did not fall below 95% of the original inoculum at *T* = 0. Adherence and invasion were similarly unaffected by storage (data not shown).

10.1128/mSphere.00647-18.2 FIG S1 Group A *Streptococcus* M75 611024 delivery characteristics and viability at −80°C. Data are means and standard deviations calculated from single experiments with four replicates. Download FIG S1, PDF file, 0.1 MB. Copyright © 2019 Osowicki et al. 2019 Osowicki et al. This content is distributed under the terms of the Creative Commons Attribution 4.0 International license.

### Antibiotic susceptibility.

M75 was susceptible to all tested antibiotics, while M12 was resistant to macrolides and fluoroquinolones (Table 2). All strains were susceptible to clindamycin, and inducible resistance was not detected.

**TABLE 2:** Antibiotic susceptibility of contemporary group A streptococcal strains M75 611024, M12 611025, and M1T1 5448

Antibiotic	Breakpoint^*a*^ (mg/liter)			Etest MIC (mg/liter)		
	S	I	R	M75	M12	5448
Penicillin	≤0.12			0.012	0.016	0.012
Erythromycin	≤0.25	0.5	≥1	0.094	16	0.125
Clindamycin	≤0.25	0.5	≥1	0.125^*b*^	0.125^*b*^	0.125^*b*^
Azithromycin	≤0.5	1	≥2	1	64	1.5
Levofloxacin	≤2	4	≥8	0.5	4	0.5
Rifampin	≤0.06		>0.5	0.064	0.064	0.125

a All CLSI breakpoints except that for rifampin (EUCAST). I, intermediate susceptibility; R, resistant; S, susceptible.

b Inducible clindamycin resistance (D test) not detected.

### Whole-genome sequencing and phylogenetic analyses.

The complete genome of M75 611024 is comprised of a single chromosome of 1,852,894 bp (Fig. 2A). M75 has the multilocus sequence type (MLST) ST150 and contains the *emm*75.0 allele and *mrp*24 and *enn*334 alleles, corresponding to the *emm*-like genes *mrp* and *enn* (P. Smeesters, personal communication, July 2018). Three putative prophage sequences were identified in M75 harboring the endonuclease streptodornase 3 (*spd3*), pyrogenic exotoxins *speL* and *speM*, and the endonuclease *sdn*. M75 shared a hypothetical ancestral relationship with a UK *emm*75 cluster (Fig. 2B), yet it represents a distinct evolving lineage, suggesting an ancestral relationship to modern-day ST150 *emm*75 clones.

One single-nucleotide polymorphism (SNP) was found for each of three M75 clones sequenced after 7 days of repeated *in vitro* passage compared to sequence of the nonpassaged parent strain. Each SNP was intergenic and different, suggestive of random mutations of unlikely functional consequence (data not shown).

M12 611025 belongs to MLST ST36 and carries the *emm*12.0 allele. It shares a high degree of genome conservation with other *emm*12 genome sequences, varying in prophage and integrative conjugative element content relative to the reference genomes HKU16 and MGAS9429 (Fig. 2C). Phylogenetic analysis alongside 141 extant *emm*12 isolates showed an evolutionary relationship with other modern ST36 strains (Fig. 2D), including recent scarlet fever outbreak strains (13).

The historical challenge strain SS-496 shares a higher degree of genetic and evolutionary similarity with the ancestral M1 reference strain SF370 relative to the modern M1T1 strains MGAS5005 and 5448 (Fig. 2E and F) (14). SS-496 contains the historical SF370-like *purA* to *nadC* genomic region encoding streptolysin O.

### Virulence factors and vaccine antigens.

M75, M12, and SS-496 carry genes for an array of adhesion and invasion factors common to many *emm* types (Table 3). M75 contains a frameshift mutation in the fibronectin binding protein Sfb1 within the FCT locus. M12 carries the streptococcal superantigen A (*ssa*) gene recently reported in scarlet fever-associated isolates in China and the United Kingdom (15). M12 does not carry the multidrug-resistant integrative conjugative element ICE-*emm*12 or the *ssa*-carrying prophage ΦHKU.vir, linked to the emergence of scarlet fever clades (13). The virulence profile of SS-496 is similar to that of pre-1980 M1 strains such as SF370, with *speH* and *speI* exotoxins and the absence of the *speA* exotoxin typical of modern isolates such as 5448 (Table 2). M75, M12, SS-496, and 5448 all possess wild-type *covRS* and *ropB* two-component virulence regulators.

**TABLE 3:** Group A *Streptococcus* virulence factor genomic screen

Gene	Function	M75 611024	M12 611025	M1T1 5448	CDC SS-496
*cfa-cfb*	CAMP factor	✓	✓	✓	✓
*tee* (*cpa*)	T-pilus antigen		✓	✓	✓
*cppA*	Putative C3-degrading proteinase	✓	✓	✓	✓
*emm*	M-protein	✓	✓	✓	✓
*endoS*	Endo-beta-*N*-acetylglucosaminidase F2 precursor	✓	✓	✓	✓
*fbp54*	Fibrinogen-binding protein	✓	✓	✓	✓
*fctA*	Major pilin Ap1 (FctA)			✓	✓
*fctB*	Minor pilin Ap2 (FctB)			✓	✓
*grab*	Protein G-related alpha 2M-binding protein		✓	✓	✓
*hasA*	HA synthase capsule	✓	✓	✓	✓
*hasB*	UDP-glucose 6-dehydrogenase capsule	✓	✓	✓	✓
*hasC*	Putative UDP-glucose pyrophosphorylase	✓	✓	✓	✓
*htrA-degP*	Serine protease	✓	✓	✓	✓
*htsA*	Putative ABC transporter periplasmic binding protein	✓	✓	✓	✓
*htsB*	Putative ABC transporter permease	✓	✓	✓	✓
*htsC*	Putative ABC transporter ATP-binding protein	✓	✓	✓	✓
*hyl*	Hyaluronoglucosaminidase	✓	✓	✓	✓
*hylA*	HA lyase precursor		✓	✓	✓
*hylP*	Hyaluronoglucosaminidase	✓	✓	✓	✓
*ideS-mac*	IgG-degrading protease	✓	✓	✓	✓
*lepA*	Signal peptidase I			✓	✓
*lmb*	Laminin binding protein	✓	✓	✓	✓
*mf-spd*	Deoxyribonuclease	✓	✓	✓	✓
*mf3*	Deoxyribonuclease	✓		✓	✓
*plr-gapA*	Glyceraldehyde-3-phosphate dehydrogenase	✓	✓	✓	✓
*prtF2*	Collagen adhesion protein		✓		
*psaA*	Manganese-binding protein	✓	✓	✓	✓
*sagA*	Streptolysin S precursor	✓	✓	✓	✓
*sclA*	Collagen-like surface protein A	✓	✓	✓	✓
*sclB*	Putative collagen-like protein			✓	✓
*scpA*	C5A peptidase precursor	✓	✓	✓	✓
*sda*	Phage-encoded streptodornase Sda		✓	✓	
*sdn*	Phage-encoded endonuclease Sdn	✓			
*sfbII-sof*	Fibronectin-binding protein	✓	✓		
*sfbX*	Fibronectin-binding protein	✓	✓		
*shp*	Hypothetical protein	✓	✓	✓	✓
*shr*	Fe^3+^-siderophore transporter	✓	✓	✓	✓
*sic*	Streptococcal inhibitor of complement			✓	
*ska*	Streptokinase precursor	✓	✓	✓	✓
*slo*	Streptolysin O	✓	✓	✓	✓
*smeZ*	Enterotoxin	✓	✓	✓	✓
*speB*	Cysteine protease	✓	✓	✓	✓
*speA*	Exotoxin A			✓	
*speG*	Exotoxin G	✓	✓	✓	✓
*speH*	Exotoxin H		✓		✓
*speI*	Exotoxin I		✓		✓
*speJ*	Exotoxin J			✓	✓
*speL*	Exotoxin L	✓			
*speM*	Exotoxin M	✓			
*spyA*	C3 family ADP-ribosyltransferase	✓	✓	✓	✓
*srtC1*	Sortase			✓	✓
*ssa*	Streptococcal superantigen A		✓		
*tig-ropA*	Trigger factor	✓	✓	✓	✓

High carriage (>60%) of protein and peptide candidate vaccine antigens was observed for M75 and M12 using a homology-based genome approach (Table 4).

**TABLE 4:** Group A *Streptococcus* candidate vaccine antigen genomic screen^*c*^

Gene/antigen	Gene identifier^*a*^	Function	M75 611024	M12 611025	M1T1 5448
		M-protein, N terminal (30-valent vaccine)	✓	✓	✓
		M-protein, C terminal (J8.0)		✓	✓
		M-protein, C terminal (StreptInCor T-cell epitope)			
		M-protein, C terminal (StreptInCor B-cell epitope)			
		M-protein, C terminal (StreptInCor common epitope)		✓	
*adi*	MGAS5005_spy1275	Arginine deaminase	✓	✓	✓
*fbaA*	MGAS5005_spy1714	Fibronectin-binding protein A			
*fbp54*	AAA57236	Fibronectin-binding protein 54	✓	✓	✓
*oppA*	M5005_spy0249	Oligopeptide-binding protein	✓	✓	✓
*GAC*	MGAS5005^*b*^	Group A carbohydrate	✓	✓	✓
*pulA*	SF370_spy1972	Putative pullulanase	✓	✓	✓
*r28*	AF091393	Rib-like cell wall protein			
*scpA*	MGAS5005_spy1715	C5a peptidase	✓	✓	✓
*sfbI*	X67947	Streptococcal fibronectin binding protein I			
*sfbII-sof*	X83303	Serum opacity factor	✓		
*shr*	SPY1530	Streptococcal hemoprotein receptor	✓	✓	✓
*sib35*	AB254157	Streptococcal immunoglobulin-binding protein 35	✓	✓	✓
*slo*	M5005_spy0124	Streptolysin O	✓	✓	✓
*spa*	MGAS8232_spyM18_2046	Streptococcal protective antigen			
*speA*	X03929	Streptococcal pyrogenic exotoxin A			✓
*speB*	M5005_spy1735	Cysteine protease	✓	✓	✓
*speC*	SF370_spy0711	Streptococcal pyrogenic exotoxin C			
*spy0651*	MGAS5005_spy0651	Cell surface protein	✓	✓	✓
*spy0762*	MGAS5005_spy0762	Hypothetical membrane associated protein	✓	✓	✓
*spy0942*	MGAS5005_spy0942	Nucleoside-binding protein	✓	✓	✓
*spyAD*	MGAS5005_spy0229	Adhesin and division protein	✓	✓	✓
*spyCEP*	MGAS5005_spy0341	Interleukin-8 serine protease	✓	✓	✓
*sse*	SF370_spy1407	Serine esterase		✓	✓
*tee*	MGAS5005_spy0109	T antigen	✓	✓	✓
*tif*	SF370_spy1612	Trigger factor	✓	✓	✓

a Nucleotide gene sequences derived from completely sequenced genomes or listed GenBank identifiers. Accession numbers for genome sequences include MGAS5005 (CP000017), SF370 (AE004092), and MGAS8232 (AE009949).

b GAC operon (∼14.2 kb) refers to MGAS5005 genome coordinates 604873 to 619151.

c BLAST analyses at a homology level of 80% for protein antigens and 100% for peptide-derived sequences.

### Human neutrophil killing assay.

M75 was most susceptible to *in vitro* killing when incubated with human neutrophils, although killing was observed for all strains (Fig. 1D).

### Mouse lethal invasive model.

Compared to M12, 5448, and SS-496, M75 was avirulent in the humanized mouse invasive disease model (Fig. 1E).

## DISCUSSION

We have described the rationale for selecting M75 for initial use in a new GAS pharyngitis CHIM in healthy adults, including results of diverse preclinical studies assessing its fitness for purpose. For context and comparison, we have presented results for three other strains (M12, 5448, and SS-496).

M75 is compatible with critical protocol points: reliable growth in an animal-free medium, retention of growth and attachment properties after prolonged storage at −80°C, consistent delivery using a commercially available swab, and susceptibility to antibiotics used to treat GAS pharyngitis. M75 looks to have an acceptable virulence profile, with the capacity to cause pharyngitis and low potential for invasive disease. M75 has attractive attachment properties for immortalized human pharyngeal and skin cell lines, with limited cellular invasion. M75 was highly susceptible to *in vitro* killing by human neutrophils, possibly due to its minimal capsular HA production. In a humanized mouse model of invasive infection M75 was avirulent, whereas M12 and both M1 strains were lethal. Whole-genome sequencing placed the strains in the context of epidemiologically related phylotypes and found broad representation of candidate vaccine antigens and a relatively restricted array of virulence factor genes in M75.

CHIM strain selection has been guided by varied general and pathogen-specific considerations, all with the goal of safely and reliably reproducing relevant and generalizable asymptomatic (infection/carriage) or symptomatic (disease) study endpoints (3, 4, 16). Suitable well-characterized strains may already exist, such as the Salmonella enterica serovar Typhi Quailes strain (17). Patients with mild to moderately severe uncomplicated disease may be a source of naturally attenuated new strains. Multiple strains, sometimes from different locations, may be required to represent natural strain diversity and/or enable heterologous rechallenge (18). If mechanisms of severe infection and/or complications are known, the implicated virulence factor(s) may be avoided (e.g., Shiga toxin-producing Escherichia coli [19] and Campylobacter jejuni inducing cross-reactive antibodies to GM1 and GQ1b gangliosides [20]). Pathogens may be modified for use, such as the propagation of single-sex Schistosoma mansoni cercariae to prevent chronic schistosomiasis (21). For vaccine studies, target antigen(s) must be present in the challenge strain(s). In every instance, strains must be characterized and be compatible with protocols for manufacturing and inoculation and with techniques to measure organism and host responses.

The limitations of this characterization effort are inherent in the rationale for pursuing a GAS CHIM. *In vitro* assays, genomics, and animal models do not fully capture or predict the dynamic elements and sequelae of human infection by GAS, a highly adapted and human-restricted pathogen. Even advanced nonhuman primate models produce a pharyngitis syndrome with important differences from human disease. A single contemporary clone, represented here by 5448, is simultaneously the most common cause in urbanized settings of both the mildest and most severe disease syndromes, with the basis for tissue tropism and bacterial-human genotype-phenotype relationships still relatively obscure. These uncertainties dictate a cautious approach extending beyond strain selection, including strain manufacture following principles of Good Manufacturing Practice, initial inclusion of healthy adults only without risk factors for severe GAS disease, a dose-ranging study to establish attack rate and safety, inpatient admission at a trials facility supported by a tertiary hospital, universal antibiotic treatment, outpatient follow-up, and echocardiography at screening and final visits.

A generic limitation of CHIM studies is the uncertain degree to which data from healthy adults experiencing a single syndrome (pharyngitis) caused by one strain (M75) can be generalized to other subjects, syndromes, strains, and settings (e.g., children with GAS skin infections due to other *emm* types in low- and middle-income countries). While inclusion of other strains and even a skin infection CHIM are conceivable extensions, model findings must be interpreted alongside knowledge derived from more naturalistic studies. For vaccine development, a GAS pharyngitis CHIM has dual scientific and strategic purposes, aiming to serve as a bridge to field trials with a more natural distribution of subjects, syndromes, and strains.

With a view to the very high priority given to participant safety and risk minimization, findings from these strain characterization studies reinforce the appropriateness of M75 for initial use in a GAS pharyngitis CHIM.

## MATERIALS AND METHODS

### Bacterial isolates.

M75 611024 and M12 611025 were isolated in 2011 from throat swabs collected from children with acute pharyngitis in Melbourne, Australia, and stored at the Murdoch Children’s Research Institute (9). Mark Walker at the University of Queensland supplied 5448 (10, 22). The U.S. Centers for Disease Control and Prevention (CDC) *Streptococcus* Laboratory provided the SS-496 strain, submitted in 1958 from Duke University.

### Growth and viability.

For administration to human volunteers, an animal-free medium must sustain sufficient strain growth. A chemically defined medium was developed (VR broth) consisting of RPMI 1640 (Gibco) and 2% (wt/vol) Veggietone genetically modified organism-free soya-peptone (Oxoid). Eight-hour growth assays were done comparing growth of M75 in this medium to that in Todd-Hewitt broth (Oxoid) with 1% (wt/vol) yeast extract (Bacto) (THY). Bacteria were grown in 125-ml Erlenmeyer flasks containing 25 ml of VR or THY broth and agitated gently at 75 rpm. To simulate manufacturing processes, M75 growth in VR broth was examined after 7 days of repeated *in vitro* passage, using frozen cultures of three postpassage clones and the prepassage parent isolate.

### HA capsule assay.

The hyaluronic acid (HA) capsule is a GAS virulence factor that resists opsonophagocytosis (23). Capsular HA levels were quantified using a test kit (Corgenix), as previously described (10).

### Attachment properties.

Cell culture lines have been used to study GAS adherence (24, 25). We used Detroit 562 (D562) human pharyngeal cells and skin HaCaT cells, simulating natural sites of infection. As previously described, total cell-associated GAS (percentage of original inoculum) and invasiveness (intracellular fraction of total cell-associated GAS) were determined using GAS grown to mid-exponential phase (optical density at 600 nm [OD_600_] of ∼0.5) in VR broth and diluted in 500 µl of assay medium (MEM with 5% fetal bovine serum; Gibco) to a multiplicity of infection of 5:1 (GAS:cells) (10). Inoculated trays were centrifuged for 5 min at 200 × *g* and incubated for 1 h at 37°C in 5% CO_2_ and then washed three times with phosphate-buffered saline (PBS) to remove nonadherent bacteria. Cell-associated GAS (adherent plus invasive) were detached using 200 µl 0.25% trypsin, lysed with 0.025% Triton-X (Sigma) in distilled water, and enumerated by track dilution on horse blood agar. To measure invasive bacteria, cells were washed once after incubation in assay medium for 1 h and then incubated for another hour in medium containing 100 µg/ml gentamicin and enumerated as before.

### Delivery characteristics.

To assess M75 viability following storage at −80°C, bacteria were grown in VR broth (OD_600_ of 0.5), centrifuged, and suspended in broth containing 10% (vol/vol) glycerol (Sanofi). Vials containing 10^5^, 10^6^, 10^7^, and 10^8^ CFU/ml were thawed at intervals and immediately tested without washing (mimicking the challenge protocol) for (i) growth in solid and liquid media, (ii) viability by enumeration, and (iii) attachment properties, as described above.

For the challenge procedure, swab uptake and release of the GAS inoculum should be consistent. We simulated direct oropharyngeal application using four Copan swabs: FLOQSwab (nylon), Dacron (polyester), and small (S) and large (L) rayon swabs. For uptake, vials of broth were weighed before and after dipping of swabs for 10 s. Swab release of GAS was measured by dipping swabs in 1-ml vials containing 1 × 10^5^ to 3 × 10^5^ CFU of M75 for 10 s, followed by transferring to 1 ml of PBS for 10 s and then enumerated by spread plate dilutions.

### Antibiotic susceptibility testing.

MICs were determined by Etest, and double disk diffusion (d-zone test) was used to detect inducible clindamycin resistance. Interpretive breakpoints of the Clinical and Laboratory Standards Institute (CLSI; penicillin, erythromycin, azithromycin, clindamycin, and levofloxacin) and the European Committee on Antimicrobial Susceptibility Testing (EUCAST; rifampin) were used (26, 27).

### Whole-genome sequencing and phylogenetic analyses.

The complete M75 611024 genome sequence was determined using long-read single-molecule real-time sequencing on the Pacific Biosciences RS II platform. Filtering of the long reads identified 104,694 reads with an average polymerase read length of 4.1 kb. A single circular assembly was generated using SMRT analysis, v2.3.0 (Pacific Biosciences), and HGAP, v3, and polished using Quiver at an average read depth of 96-fold. To aid in assembly validation, M75 was also sequenced on an Illumina Next-seq 500 to produce paired-end reads with a read length of 150 bases. The M75 611024 genome sequence has been submitted to GenBank (accession number CP033621). The genomes of M12 611025 and CDC SS-496 were sequenced by Illumina Next-seq 500 with a paired-end read length of 150 bases. Draft genome assemblies were generated using SPAdes v3.12.0. Illumina short reads of M12 611025 (accession number SRR8217179) and CDC SS-496 (SRR8217180) have been submitted to the Short Read Archive (PRJNA504701).

To study M75 genomic stability, three clones were sequenced by Illumina Next-seq 500, with 150-bp paired-end reads, after 7 days of repeated *in vitro* passage. These sequences were aligned with the prepassage parent M75 reference sequence to identify single-nucleotide polymorphisms (SNPs).

Phylogenetic analysis of a global data set of *emm*75 isolates was determined by mapping short read sequences of 131 global *emm*75 genomes from the United Kingdom (*n* = 124), United States (*n* = 4), and France (*n* = 3) (28–30) to the M75 611024 reference genome with BWA MEM (v0.7.16). SNPs with a Phred quality score of ≥30 were identified in each isolate using SAMtools pileup with a minimum coverage of 30×. Prophage sequences within M75 611024 were identified using the Phaster server, with SNPs located within these prophage excluded, as they represent evolutionary confounders. A maximum likelihood phylogenetic tree was built from 1,046 concatenated SNP sites using RAxML, v8.2.8, with the general time-reversible model and gamma correction with 100 bootstrap resamplings to assess phylogenetic support.

Phylogeny of 141 *emm*12 genomes, including the Illumina reads of M12 611025 and sequences from Australia, the United States, Hong Kong, and mainland China, was analyzed by mapping to the reference genome HKU16 (strain QMH11M0907901 [GenBank accession no. AFRY01000001]) from 1,452 vertically inherited SNPs as previously described (13). Illumina reads of the M1 genome sequence CDC SS-496 were mapped to MGAS5005 (GenBank accession no. NC_007297) and other M1 reference genomes with phylogeny inferred form 780 vertically inherited SNPs.

### Virulence factors and vaccine antigens.

Virulence gene carriage was determined for M75, M12, 5448, and SS-496 by blastN screening assemblies against the virulence factor database (VFDB) (31). Gene presence was defined by an 80% nucleotide cutoff over 80% of the gene length.

Protection in animal models has been shown for more than twenty-five candidate GAS vaccine protein antigens and several peptide-based antigens (32). For protein antigens, presence was defined by an 80% nucleotide cutoff over 80% of the gene length. For sequence-constrained peptide-based vaccine epitopes J8.0 (SREAKKQVEKAL) (33) as well as the StreptInCor sequence (KGLRRDLDASREAKKQLEAEQQKLEEQNKISEASRKGLRRDLDASREAKKQVEKA) (34) and associated T-cell (KGLRRDLDASREAKKQLEAEQQ), B-cell (ASRKGLRRDLDASREAKKQVEKA), and common B-T-cell (KGLRRDLDASREAKKQ) epitopes, a 100% nucleotide sequence match was taken to define presence, although 100% homology may not be required to induce production of broadly cross-reactive antibodies and vaccine protection.

### Neutrophil killing assay.

Survival of M75, M12, and 5448 incubated with human neutrophils *in vitro* was assayed as previously described (35). Experiments were performed in triplicate using mid-exponential-phase GAS at a multiplicity of infection of 10:1. Differences in neutrophil survival were analyzed using 1-way analysis of variance (GraphPad Prism).

### Murine invasive model.

Strain virulence was compared in a humanized plasminogen transgenic *AlbPLG1* mouse model (36). In separate experiments, groups (*n* = 10) of *AlbPLG1^+/−^* mice were administered subcutaneous doses of either M75 (7 × 10^7^ CFU), M12 (8 × 10^7^ CFU), 5448 (5 × 10^7^ CFU), or SS-496 (3 × 10^7^ CFU), and survival was monitored for 10 days, as previously described (37, 38).

### Ethics statement.

Animal procedures followed the *Australian Code for the Care and Use of Animals for Scientific Purposes* and were approved by the University of Queensland Animal Ethics Committee (SCMB/140/16/NHMRC) (39). An initial dose-ranging CHIM study has been approved by The Alfred Hospital Ethics Committee (500/17) and is registered at ClinicalTrials.gov (NCT03361163).

### Data availability.

The M75 611024 genome sequence has been submitted to GenBank (accession number CP033621). Illumina short reads of M12 611025 (accession number SRR8217179) and CDC SS-496 (SRR8217180) have been submitted to the Short Read Archive (PRJNA504701). M75 was also sequenced on an Illumina Next-seq 500 to produce paired-end reads with a read length of 150 bases (accession number SRR8217178). 

## References

[1] Walker MJ, Barnett TC, McArthur JD, Cole JN, Gillen CM, Henningham A, Sriprakash KS, Sanderson-Smith ML, Nizet V 2014 Disease manifestations and pathogenic mechanisms of group A Streptococcus. Clin Microbiol Rev 27:264–301. doi:10.1128/CMR.00101-13. 24696436 PMC3993104

[2] Steer AC, Carapetis JR, Dale JB, Fraser JD, Good MF, Guilherme L, Moreland NJ, Mulholland EK, Schodel F, Smeesters PR 2016 Status of research and development of vaccines for Streptococcus pyogenes. Vaccine 34:2953–2958. doi:10.1016/j.vaccine.2016.03.073. 27032515

[3] Roestenberg M, Hoogerwerf MA, Ferreira DM, Mordmuller B, Yazdanbakhsh M 2018 Experimental infection of human volunteers. Lancet Infect Dis 18:e312–e322. doi:10.1016/S1473-3099(18)30177-4. 29891332

[4] The Academy of Medical Sciences. 2018 Controlled human infection model studies: summary of a workshop held on 6 February 2018. The Academy of Medical Sciences, London, United Kingdom.

[5] Polly SM, Waldman RH, High P, Wittner MK, Dorfman A, Fox EN 1975 Protective studies with a group A streptococcal M protein vaccine. II. Challenge of volunteers after local immunization in the upper respiratory tract. J Infect Dis 131:217–224. doi:10.1093/infdis/131.3.217. 1092765

[6] D'Alessandri R, Plotkin G, Kluge RM, Wittner MK, Fox EN, Dorfman A, Waldman RH 1978 Protective studies with group A streptococcal M protein vaccine. III. Challenge of volunteers after systemic or intranasal immunization with type 3 or type 12 group A Streptococcus. J Infect Dis 138:712–718. doi:10.1093/infdis/138.6.712. 368261

[7] Fox EN, Waldman RH, Wittner MK, Mauceri AA, Dorfman A 1973 Protective study with a group A streptococcal M protein vaccine. Infectivity challenge of human volunteers. J Clin Investig 52:1885–1892. doi:10.1172/JCI107372. 4719668 PMC302470

[8] Osowicki J, Vekemans J, Kaslow DC, Friede MH, Kim JH, Steer AC 2018 WHO/IVI global stakeholder consultation on group A Streptococcus vaccine development: report from a meeting held on 12-13 December 2016. Vaccine 36:3397–3405. doi:10.1016/j.vaccine.2018.02.068. 29496349

[9] Dunne EM, Marshall JL, Baker CA, Manning J, Gonis G, Danchin MH, Smeesters PR, Satzke C, Steer AC 2013 Detection of group a streptococcal pharyngitis by quantitative PCR. BMC Infect Dis 13:312. doi:10.1186/1471-2334-13-312. 23844865 PMC3711935

[10] Hollands A, Pence MA, Timmer AM, Osvath SR, Turnbull L, Whitchurch CB, Walker MJ, Nizet V 2010 Genetic switch to hypervirulence reduces colonization phenotypes of the globally disseminated group A streptococcus M1T1 clone. J Infect Dis 202:11–19. doi:10.1086/653124. 20507231 PMC2880657

[11] Bessen DE, Smeesters PR, Beall BW 2018 Molecular epidemiology, ecology, and evolution of group A streptococci. Microbiol Spectr 6:CPP3-0009-21018. doi:10.1128/microbiolspec.CPP3-0009-2018. PMC11633622 30191802

[12] Cole JN, Pence MA, von Kockritz-Blickwede M, Hollands A, Gallo RL, Walker MJ, Nizet V 2010 M protein and hyaluronic acid capsule are essential for in vivo selection of covRS mutations characteristic of invasive serotype M1T1 group A Streptococcus. mBio 1:e00191-10. doi:10.1128/mBio.00191-10. 20827373 PMC2934611

[13] Davies MR, Holden MT, Coupland P, Chen JH, Venturini C, Barnett TC, Zakour NL, Tse H, Dougan G, Yuen KY, Walker MJ 2015 Emergence of scarlet fever Streptococcus pyogenes emm12 clones in Hong Kong is associated with toxin acquisition and multidrug resistance. Nat Genet 47:84–87. doi:10.1038/ng.3147. 25401300

[14] Nasser W, Beres SB, Olsen RJ, Dean MA, Rice KA, Long SW, Kristinsson KG, Gottfredsson M, Vuopio J, Raisanen K, Caugant DA, Steinbakk M, Low DE, McGeer A, Darenberg J, Henriques-Normark B, Van Beneden CA, Hoffmann S, Musser JM 2014 Evolutionary pathway to increased virulence and epidemic group A Streptococcus disease derived from 3,615 genome sequences. Proc Natl Acad Sci U S A 111:E1768–E1776. doi:10.1073/pnas.1403138111. 24733896 PMC4035937

[15] You Y, Davies MR, Protani M, McIntyre L, Walker MJ, Zhang J 2018 Scarlet fever epidemic in China caused by Streptococcus pyogenes serotype M12: epidemiologic and molecular analysis. EBioMedicine 28:128–135. doi:10.1016/j.ebiom.2018.01.010. 29342444 PMC5835554

[16] Darton TC, Blohmke CJ, Moorthy VS, Altmann DM, Hayden FG, Clutterbuck EA, Levine MM, Hill AV, Pollard AJ 2015 Design, recruitment, and microbiological considerations in human challenge studies. Lancet Infect Dis 15:840–851. doi:10.1016/S1473-3099(15)00068-7. 26026195

[17] Waddington CS, Darton TC, Jones C, Haworth K, Peters A, John T, Thompson BA, Kerridge SA, Kingsley RA, Zhou L, Holt KE, Yu LM, Lockhart S, Farrar JJ, Sztein MB, Dougan G, Angus B, Levine MM, Pollard AJ 2014 An outpatient, ambulant-design, controlled human infection model using escalating doses of Salmonella Typhi challenge delivered in sodium bicarbonate solution. Clin Infect Dis 58:1230–1240. doi:10.1093/cid/ciu078. 24519873 PMC3982839

[18] Stanisic DI, McCarthy JS, Good MF 2018 Controlled human malaria infection: applications, advances, and challenges. Infect Immun 86:e00479-17. doi:10.1128/IAI.00479-17. 28923897 PMC5736798

[19] Harro C, Chakraborty S, Feller A, DeNearing B, Cage A, Ram M, Lundgren A, Svennerholm AM, Bourgeois AL, Walker RI, Sack DA 2011 Refinement of a human challenge model for evaluation of enterotoxigenic Escherichia coli vaccines. Clin Vaccine Immunol 18:1719–1727. doi:10.1128/CVI.05194-11. 21852546 PMC3187035

[20] Tribble DR, Baqar S, Carmolli MP, Porter C, Pierce KK, Sadigh K, Guerry P, Larsson CJ, Rockabrand D, Ventone CH, Poly F, Lyon CE, Dakdouk S, Fingar A, Gilliland T, Daunais P, Jones E, Rymarchyk S, Huston C, Darsley M, Kirkpatrick BD 2009 Campylobacter jejuni strain CG8421: a refined model for the study of Campylobacteriosis and evaluation of Campylobacter vaccines in human subjects. Clin Infect Dis 49:1512–1519. doi:10.1086/644622. 19842970

[21] Janse JJ, Langenberg MCC, Kos-Van Oosterhoud J, Ozir-Fazalalikhan A, Brienen EAT, Winkel BMF, Erkens MAA, van der Beek MT, van Lieshout L, Smits HH, Webster BL, Zandvliet ML, Verbeek R, Westra IM, Meij P, Visser LG, van Diepen A, Hokke CH, Yazdanbakhsh M, Roestenberg M 2018 Establishing the production of male Schistosoma mansoni Cercariae for a controlled human infection model. J Infect Dis 218:1142–1146. doi:10.1093/infdis/jiy275. 29905805

[22] Chatellier S, Ihendyane N, Kansal RG, Khambaty F, Basma H, Norrby-Teglund A, Low DE, McGeer A, Kotb M 2000 Genetic relatedness and superantigen expression in group A streptococcus serotype M1 isolates from patients with severe and nonsevere invasive diseases. Infect Immun 68:3523–3534. doi:10.1128/IAI.68.6.3523-3534.2000. 10816507 PMC97638

[23] Dale JB, Washburn RG, Marques MB, Wessels MR 1996 Hyaluronate capsule and surface M protein in resistance to opsonization of group A streptococci. Infect Immun 64:1495–1501. 8613352 10.1128/iai.64.5.1495-1501.1996 PMC173953

[24] Loh JMS, Tsai JC, Proft T 2017 The ability of group A Streptococcus to adhere to immortalized human skin versus throat cell lines does not reflect their predicted tissue tropism. Clin Microbiol Infect 23:677. 28336383 10.1016/j.cmi.2017.03.011

[25] Ryan PA, Juncosa B 2016 Group A streptococcal adherence *In* Ferretti JJ, Stevens DL, Fischetti VA (ed), Streptococcus pyogenes: basic biology to clinical manifestations. University of Oklahoma Health Sciences Center, Oklahoma City, OK. 26866208

[26] Clinical and Laboratory Standards Institute. 2016 Performance standards for antimicrobial susceptibility testing: twenty-sixth informational supplement M100-S26. CLSI, Wayne, PA.

[27] The European Committee on Antimicrobial Susceptibility Testing. 2019 Breakpoint tables for interpretation of MICs and zone diameters, version 9.0. http://www.eucast.org/fileadmin/src/media/PDFs/EUCAST_files/Breakpoint_tables/v_9.0_Breakpoint_Tables.pdf.

[28] Kapatai G, Coelho J, Platt S, Chalker VJ 2017 Whole genome sequencing of group A Streptococcus: development and evaluation of an automated pipeline for emm gene typing. Peer J 5:e3226. doi:10.7717/peerj.3226. 28462035 PMC5410157

[29] Chochua S, Metcalf BJ, Li Z, Rivers J, Mathis S, Jackson D, Gertz RE Jr, Srinivasan V, Lynfield R, Van Beneden C, McGee L, Beall B 2017 Population and whole genome sequence based characterization of invasive group A streptococci recovered in the United States during 2015. mBio 8:e01422-17. doi:10.1128/mBio.01422-17. 28928212 PMC5605940

[30] Rochefort A, Boukthir S, Moullec S, Meygret A, Adnani Y, Lavenier D, Faili A, Kayal S 2017 Full sequencing and genomic analysis of three emm75 group A Streptococcus strains recovered in the course of an epidemiological shift in French Brittany. Genome Announc 5:e00957-17. doi:10.1128/genomeA.00957-17. 28963207 PMC5624753

[31] Chen L, Zheng D, Liu B, Yang J, Jin Q 2016 VFDB 2016: hierarchical and refined dataset for big data analysis–10 years on. Nucleic Acids Res 44:D694–D697. doi:10.1093/nar/gkv1239. 26578559 PMC4702877

[32] Henningham A, Gillen CM, Walker MJ 2013 Group a streptococcal vaccine candidates: potential for the development of a human vaccine. Curr Top Microbiol Immunol 368:207–242. doi:10.1007/82_2012_284. 23250780

[33] Batzloff MR, Hayman WA, Davies MR, Zeng M, Pruksakorn S, Brandt ER, Good MF 2003 Protection against group A streptococcus by immunization with J8-diphtheria toxoid: contribution of J8- and diphtheria toxoid-specific antibodies to protection. J Infect Dis 187:1598–1608. doi:10.1086/374800. 12721940

[34] Guilherme L, Fae KC, Higa F, Chaves L, Oshiro SE, Freschi de Barros S, Puschel C, Juliano MA, Tanaka AC, Spina G, Kalil J 2006 Towards a vaccine against rheumatic fever. Clin Dev Immunol 13:125–132. doi:10.1080/17402520600877026. 17162355 PMC2270766

[35] Buchanan JT, Simpson AJ, Aziz RK, Liu GY, Kristian SA, Kotb M, Feramisco J, Nizet V 2006 DNase expression allows the pathogen group A Streptococcus to escape killing in neutrophil extracellular traps. Curr Biol 16:396–400. doi:10.1016/j.cub.2005.12.039. 16488874

[36] Sun H, Ringdahl U, Homeister JW, Fay WP, Engleberg NC, Yang AY, Rozek LS, Wang X, Sjobring U, Ginsburg D 2004 Plasminogen is a critical host pathogenicity factor for group A streptococcal infection. Science 305:1283–1286. doi:10.1126/science.1101245. 15333838

[37] Walker MJ, Hollands A, Sanderson-Smith ML, Cole JN, Kirk JK, Henningham A, McArthur JD, Dinkla K, Aziz RK, Kansal RG, Simpson AJ, Buchanan JT, Chhatwal GS, Kotb M, Nizet V 2007 DNase Sda1 provides selection pressure for a switch to invasive group A streptococcal infection. Nat Med 13:981–985. doi:10.1038/nm1612. 17632528

[38] Maamary PG, Sanderson-Smith ML, Aziz RK, Hollands A, Cole JN, McKay FC, McArthur JD, Kirk JK, Cork AJ, Keefe RJ, Kansal RG, Sun H, Taylor WL, Chhatwal GS, Ginsburg D, Nizet V, Kotb M, Walker MJ 2010 Parameters governing invasive disease propensity of non-M1 serotype group A streptococci. J Innate Immun 2:596–606. doi:10.1159/000317640. 20814186 PMC3219504

[39] National Health and Medical Research Council. 2013 Australian code for the care and use of animals for scientific purposes, 8th ed National Health and Medical Research Council, Canberra, Australia https://nhmrc.gov.au/about-us/publications/australian-code-care-and-use-animals-scientific-purposes.

[40] Alikhan NF, Petty NK, Ben Zakour NL, Beatson SA 2011 BLAST Ring Image Generator (BRIG): simple prokaryote genome comparisons. BMC Genomics 12:402. doi:10.1186/1471-2164-12-402. 21824423 PMC3163573

[41] O'Loughlin RE, Roberson A, Cieslak PR, Lynfield R, Gershman K, Craig A, Albanese BA, Farley MM, Barrett NL, Spina NL, Beall B, Harrison LH, Reingold A, Van Beneden C 2007 The epidemiology of invasive group A streptococcal infection and potential vaccine implications: United States, 2000-2004. Clin Infect Dis 45:853–862. doi:10.1086/521264. 17806049

[42] Nelson GE, Pondo T, Toews KA, Farley MM, Lindegren ML, Lynfield R, Aragon D, Zansky SM, Watt JP, Cieslak PR, Angeles K, Harrison LH, Petit S, Beall B, Van Beneden CA 2016 Epidemiology of invasive group A streptococcal infections in the United States, 2005-2012. Clin Infect Dis 63:478–486. doi:10.1093/cid/ciw248. 27105747 PMC5776658

