# Supplementary material for: A Controlled Human Infection Model of Group A Streptococcus Pharyngitis: Which Strain and Why?
Source: mSphere. 2019 Feb 13;4(1):e00647-18. doi: 10.1128/mSphere.00647-18 (PMC6374595; doi:10.1128/mSphere.00647-18)
Supplement: TABLE S1 [file mSphere.00647-18-st001.pdf]

Table S1

Desirable strain characteristics	GAS M75 611024

A definite but uncommon contemporary cause of symptomatic pharyngitis

- Isolated in 2011 from the throat of a child with pharyngitis in Melbourne(1)
- Pre-existing immunity in adults is unknown (no correlate of protection)
- *emm*75 <5% of global GAS isolates (all sites and settings)(2)
- *emm*75 strains in selected GAS pharyngitis series since 2000

Location	n	*emm*75
Fiji(3)	85	1.2%
Northern Territory, Australia(4)	635; 235 pharyngeal isolates	1.3%
Denmark(5)	352	2.5%
Chile(6)	80	2.5%
Mali(7)	396	2.5%
New Zealand(8)	349	2.6%
Greece(9)	1282; 1080 pharyngeal isolates	2.7%
Japan(10)	332	2.7%
France(11)	585	2.9%
Canada(12)	1434	3.1%
Ethiopia(13)	155	3.2%
USA(12)	7040	4.7%
Cape Town, South Africa(14)	157	7.6%
Germany(15)	216	7.9%
Tunisia(16)	43	32.5%

Should cause skin infection

- E pattern 'generalist' (throat & skin infections)(17)
- Cluster E6 - linked phylogenetically to D pattern skin isolates(17, 18)

An uncommon cause of invasive GAS disease and immunological sequelae

- *emm*75 strains very rarely associated with ARF/RHD or APSGN(19-26)
- *emm*75 strains in selected invasive GAS series since 2000

Location	n	*emm*75
Portugal (27)	160	0
Northern Territory, Australia (28)	82	0
France (11)	125	0.8%
Alberta, Canada (29)	3105	1%
Finland (30)	1122	1.2%
Kilifi, Kenya (31)	357	1.4%
Denmark (5)	201	1.5%
Ireland (32)	473	1.5%
Fiji (33)	55	1.8%
Sydney, Australia (34)	55	1.8%
Scotland (35)	357	2%
USA (36, 37)*	17,002	2.4%
Norway (38)	756	2.1%
Japan (10)	74	4.1%
Thailand (39)	234	5.1%

*CDC Active Bacterial Core surveillance 2000-2016 (Chris A. Van Beneden, Personal Communication, September 11th, 2018)

Should have predictable and limited virulence, and be suitable for use in animal models

- covR/S virulence regulator wildtype (non-mutant)
- Does not bind plasminogen and fibrinogen(18)
- *emm*75 strains have been used in murine models of nasopharyngitis and invasive disease, and have been successfully transformed for bioluminescence(40)

Should have limited antibiotic resistance

- See main text

The challenge strain should possess a wide array of candidate vaccine antigens

- See main text

ARF: acute rheumatic fever, APSGN: acute post-streptococcal glomerulonephritis, GAS: group A *Streptococcus*; RHD: rheumatic heart disease

References for Supplemental Table S1

1. Dunne EM, Marshall JL, Baker CA, Manning J, Gonis G, Danchin MH, Smeesters PR, Satzke C, Steer AC. 2013. Detection of group a streptococcal pharyngitis by quantitative PCR. BMC Infect Dis 13:312.
2. Steer AC, Law I, Matatolu L, Beall BW, Carapetis JR. 2009. Global emm type distribution of group A streptococci: systematic review and implications for vaccine development. Lancet Infect Dis 9:611-6.
3. Steer AC, Magor G, Jenney AW, Kado J, Good MF, McMillan D, Batzloff M, Carapetis JR. 2009. emm and C-repeat region molecular typing of beta-hemolytic Streptococci in a tropical country: implications for vaccine development. J Clin Microbiol 47:2502-9.
4. Richardson LJ, Towers RJ, Cheng AC, Currie BJ, Carapetis JR, Giffard PM, McDonald MI. 2010. Diversity of emm sequence types in group A beta-haemolytic streptococci in two remote Northern Territory Indigenous communities: implications for vaccine development. Vaccine 28:5301-5.
5. Ekelund K, Darenberg J, Norrby-Teglund A, Hoffmann S, Bang D, Skinhoj P, Konradsen HB. 2005. Variations in emm type among group A streptococcal isolates causing invasive or noninvasive infections in a nationwide study. J Clin Microbiol 43:3101-9.
6. Wozniak A, Rojas P, Rodriguez C, Undabarrena A, Garate C, Riedel I, Roman JC, Kalergis AM, Garcia P. 2012. M-protein gene-type distribution and hyaluronic acid capsule in group A Streptococcus clinical isolates in Chile: association of emm gene markers with csrR alleles. Epidemiol Infect 140:1286-95.
7. Tapia MD, Sow SO, Tamboura B, Keita MM, Berthe A, Samake M, Nataro JP, Onwuchekwa UO, Penfound TA, Blackwelder W, Dale JB, Kotloff KL. 2015. Streptococcal pharyngitis in schoolchildren in Bamako, Mali. Pediatr Infect Dis J 34:463-8.
8. Williamson DA, Smeesters PR, Steer AC, Morgan J, Davies M, Carter P, Upton A, Tong SY, Fraser J, Moreland NJ. 2016. Comparative M-protein analysis of Streptococcus pyogenes from pharyngitis and skin infections in New Zealand: Implications for vaccine development. BMC Infect Dis 16:561.
9. Koutouzi F, Tsakris A, Chatzichristou P, Koutouzis E, Daikos GL, Kirikou E, Petropoulou N, Syriopoulou V, Michos A. 2015. Streptococcus pyogenes emm Types and Clusters during a 7-Year Period (2007 to 2013) in Pharyngeal and Nonpharyngeal Pediatric Isolates. J Clin Microbiol 53:2015-21.
10. Wajima T, Murayama SY, Sunaoshi K, Nakayama E, Sunakawa K, Ubukata K. 2008. Distribution of emm type and antibiotic susceptibility of group A streptococci causing invasive and noninvasive disease. J Med Microbiol 57:1383-8.
11. d'Humieres C, Bidet P, Levy C, Bechet S, Bonacorsi S, Bingen E, Cohen R. 2015. Comparative epidemiology of Streptococcus pyogenes emm-types causing invasive and noninvasive infections in French children by use of high-resolution melting-polymerase chain reaction. Pediatr Infect Dis J 34:557-61.
12. Shulman ST, Tanz RR, Dale JB, Beall B, Kabat W, Kabat K, Cederlund E, Patel D, Rippe J, Li Z, Sakota V, North American Streptococcal Pharyngitis Surveillance G. 2009. Seven-year surveillance of north american pediatric group a streptococcal pharyngitis isolates. Clin Infect Dis 49:78-84.
13. Tewodros W, Kronvall G. 2005. M protein gene (emm type) analysis of group A beta-hemolytic streptococci from Ethiopia reveals unique patterns. J Clin Microbiol 43:4369-76.
14. Engel ME, Muhamed B, Whitelaw AC, Musvosvi M, Mayosi BM, Dale JB. 2014. Group A streptococcal emm type prevalence among symptomatic children in Cape Town and potential vaccine coverage. Pediatr Infect Dis J 33:208-10.
15. Brandt CM, Spellerberg B, Honscha M, Truong ND, Hoevener B, Lutticken R. 2001. Typing of Streptococcus pyogenes strains isolated from throat infections in the region of Aachen, Germany. Infection 29:163-5.
16. Mzoughi R, Bouallegue O, Selmi H, Ben Said H, Essoussi AS, Jeddi M. 2004. Group A streptococci in children with acute pharyngitis in Sousse, Tunisia. East Mediterr Health J 10:488-93.
17. Bessen DE. 2016. Tissue tropisms in group A Streptococcus: what virulence factors distinguish pharyngitis from impetigo strains? Curr Opin Infect Dis 29:295-303.
18. Sanderson-Smith M, De Oliveira DM, Guglielmini J, McMillan DJ, Vu T, Holien JK, Henningham A, Steer AC, Bessen DE, Dale JB, Curtis N, Beall BW, Walker MJ, Parker MW, Carapetis JR, Van Melderen L, Sriprakash KS, Smeesters PR, Group MPS. 2014. A systematic and functional

classification of Streptococcus pyogenes that serves as a new tool for molecular typing and vaccine development. J Infect Dis 210:1325-38.
19. Williamson DA, Smeesters PR, Steer AC, Steemson JD, Ng AC, Proft T, Fraser JD, Baker MG, Morgan J, Carter PE, Moreland NJ. 2015. M-Protein Analysis of Streptococcus pyogenes Isolates Associated with Acute Rheumatic Fever in New Zealand. J Clin Microbiol 53:3618-20.
20. Erdem G, Mizumoto C, Esaki D, Reddy V, Kurahara D, Yamaga K, Abe L, Johnson D, Yamamoto K, Kaplan EL. 2007. Group A streptococcal isolates temporally associated with acute rheumatic fever in Hawaii: differences from the continental United States. Clin Infect Dis 45:e20-4.
21. Parks T, Smeesters PR, Steer AC. 2012. Streptococcal skin infection and rheumatic heart disease. Curr Opin Infect Dis 25:145-53.
22. Walker MJ, Barnett TC, McArthur JD, Cole JN, Gillen CM, Henningham A, Sriprakash KS, Sanderson-Smith ML, Nizet V. 2014. Disease manifestations and pathogenic mechanisms of Group A Streptococcus. Clin Microbiol Rev 27:264-301.
23. Chaturvedi S, Boyd R, Krause V. 2018. Acute Post-Streptococcal Glomerulonephritis in the Northern Territory of Australia: A Review of Data from 2009 to 2016 and Comparison with the Literature. Am J Trop Med Hyg 99:1643-1648.
24. Speers DJ, Levy A, Gichamo A, Eastwood A, Leung MJ. 2017. M protein gene (emm type) analysis of group A Streptococcus isolates recovered during an acute glomerulonephritis outbreak in northern Western Australia. Pathology 49:765-769.
25. Marshall CS, Cheng AC, Markey PG, Towers RJ, Richardson LJ, Fagan PK, Scott L, Krause VL, Currie BJ. 2011. Acute post-streptococcal glomerulonephritis in the Northern Territory of Australia: a review of 16 years data and comparison with the literature. Am J Trop Med Hyg 85:703-10.
26. Zheng MH, Jiao ZQ, Zhang LJ, Yu SJ, Tang GP, Yan XM, He LH, Meng FL, Zhao F, Zhang MJ, Xiao D, Yang YH, Nie W, Zhang JZ, Wang ZJ. 2009. Genetic analysis of group A streptococcus isolates recovered during acute glomerulonephritis outbreaks in Guizhou Province of China. J Clin Microbiol 47:715-20.
27. Friaes A, Pinto FR, Silva-Costa C, Ramirez M, Melo-Cristino J. 2012. Group A streptococci clones associated with invasive infections and pharyngitis in Portugal present differences in emm types, superantigen gene content and antimicrobial resistance. BMC Microbiol 12:280.
28. Boyd R, Patel M, Currie BJ, Holt DC, Harris T, Krause V. 2016. High burden of invasive group A streptococcal disease in the Northern Territory of Australia. Epidemiol Infect 144:1018-27.
29. Tyrrell GJ, Fathima S, Kakulphimp J, Bell C. 2018. Increasing Rates of Invasive Group A Streptococcal Disease in Alberta, Canada; 2003-2017. Open Forum Infect Dis 5:ofy177.
30. Smit PW, Lindholm L, Lyytikainen O, Jalava J, Patari-Sampo A, Vuopio J. 2015. Epidemiology and emm types of invasive group A streptococcal infections in Finland, 2008-2013. Eur J Clin Microbiol Infect Dis 34:2131-6.
31. Seale AC, Davies MR, Anampiu K, Morpeth SC, Nyongesa S, Mwarumba S, Smeesters PR, Efstratiou A, Karugutu R, Mturi N, Williams TN, Scott JA, Kariuki S, Dougan G, Berkley JA. 2016. Invasive Group A Streptococcus Infection among Children, Rural Kenya. Emerg Infect Dis 22:224-32.
32. Meehan M, Murchan S, Gavin PJ, Drew RJ, Cunney R. 2018. Epidemiology of an upsurge of invasive group A streptococcal infections in Ireland, 2012-2015. J Infect 77:183-190.
33. Steer AC, Jenney A, Kado J, Good MF, Batzloff M, Waqatakirewa L, Mullholland EK, Carapetis JR. 2009. Prospective surveillance of invasive group a streptococcal disease, Fiji, 2005-2007. Emerg Infect Dis 15:216-22.
34. Sivagnanam S, Zhou F, Lee AS, O'Sullivan MV. 2015. Epidemiology of invasive group A Streptococcus infections in Sydney, Australia. Pathology 47:365-71.
35. Lindsay DS, Brown AW, Scott KJ, Denham B, Thom L, Rundell G, Ure R, Jones B, Smith AJ. 2016. Circulating emm types of Streptococcus pyogenes in Scotland: 2011-2015. J Med Microbiol 65:1229-1231.
36. O'Loughlin RE, Roberson A, Cieslak PR, Lynfield R, Gershman K, Craig A, Albanese BA, Farley MM, Barrett NL, Spina NL, Beall B, Harrison LH, Reingold A, Van Beneden C. 2007. The epidemiology of invasive group A streptococcal infection and potential vaccine implications: United States, 2000-2004. Clin Infect Dis 45:853-62.

37. Nelson GE, Pondo T, Toews KA, Farley MM, Lindegren ML, Lynfield R, Aragon D, Zansky SM, Watt JP, Cieslak PR, Angeles K, Harrison LH, Petit S, Beall B, Van Beneden CA. 2016. Epidemiology of Invasive Group A Streptococcal Infections in the United States, 2005-2012. Clin Infect Dis 63:478-86.
38. Naseer U, Steinbakk M, Blystad H, Caugant DA. 2016. Epidemiology of invasive group A streptococcal infections in Norway 2010-2014: A retrospective cohort study. Eur J Clin Microbiol Infect Dis 35:1639-48.
39. Paveenkittiporn W, Nozawa T, Dejsirilert S, Nakagawa I, Hamada S. 2016. Prevalent emm types and superantigen gene patterns of group A Streptococcus in Thailand. Epidemiol Infect 144:864-9.
40. Alam FM, Bateman C, Turner CE, Wiles S, Sriskandan S. 2013. Non-invasive monitoring of Streptococcus pyogenes vaccine efficacy using biophotonic imaging. PLoS One 8:e82123.
