# Supplementary figures and images for: A Controlled Human Infection Model of Group A Streptococcus Pharyngitis: Which Strain and Why?
Source: mSphere. 2019 Feb 13;4(1):e00647-18. doi: 10.1128/mSphere.00647-18 (PMC6374595; doi:10.1128/mSphere.00647-18)

Figure S1

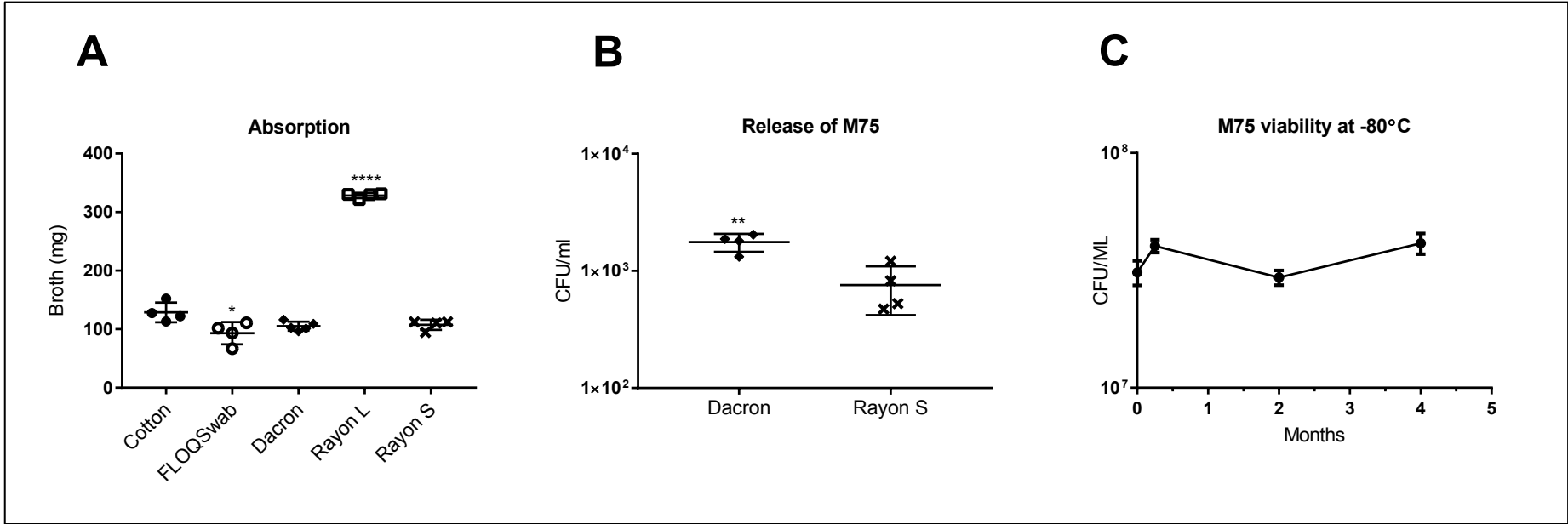

Supplement: FIG S1 [file mSphere.00647-18-sf001.pdf]
